# Supplementary material for: Polyketide synthase gene clusters in the ericoid mycorrhizal fungus Oidiodendron maius: genome-wide prediction, expression profiling, and functional characterization of knockout mutants
Source: Front Plant Sci. 2026 Sep 16;17:1924430. doi: 10.3389/fpls.2026.1924430 (PMC13623903; doi:10.3389/fpls.2026.1924430)
Supplement: Supplementary file 1 [file SupplementaryFile1.zip › Supplementary Materials/Supplementary Folder 1.zip!/Oidma1_AssemblyScaffolds_Repeatmasked/knownclusterblast/cluster36/jgi.p_Oidma1_44457_mibig_hits.html]

| MIBiG Protein | Description | MIBiG Cluster | MiBiG Product | % ID | % Coverage | BLAST Score | E-value |
| --- | --- | --- | --- | --- | --- | --- | --- |
| EFY94502.1 | aldo-keto\_reductase | BGC0000337 | Destruxins\_biosynthetic\_gene\_cluster | 47 | 100.0 | 282 | 5.2e-76 |
| AGC09504.1 | LobA6 | BGC0001183 | Lobophorin\_biosynthetic\_gene\_cluster | 35 | 97.4358974359 | 171 | 1.7e-42 |
| AGI99477.1 | aldo/keto\_reductase | BGC0001004 | Lobophorin\_biosynthetic\_gene\_cluster | 35 | 97.4358974359 | 171 | 1.7e-42 |
| ACB46464.1 | unknown | BGC0000082 | Kijanimicin\_biosynthetic\_gene\_cluster | 42 | 50.7122507123 | 133 | 6.6e-31 |
| ABG00042.1 | aldo/keto\_reductase | BGC0000387 | Mangotoxin\_biosynthetic\_gene\_cluster | 35 | 51.566951567 | 104 | 3.3e-22 |
| WP\_030269246.1 | aldo/keto\_reductase | BGC0001308 | Simocyclinone\_D9\_/\_simocyclinone\_D10\_/\_simocyclinone\_D11 | 28 | 94.5868945869 | 95 | 1.2e-19 |
| AAU93802.1 | deoxyhexose\_reductase | BGC0000054 | Erythromycin\_biosynthetic\_gene\_cluster | 31 | 61.5384615385 | 94 | 3.4e-19 |
| BAC76487.1 | putative\_NDP-4-keto-2,6-dideoxyhexose\_2,3-enoyl\_reductase | BGC0000085 | Lankamycin\_biosynthetic\_gene\_cluster | 31 | 61.5384615385 | 86 | 5.4e-17 |
| AAG13914.1 | TDP-4-keto-6-deoxyhexose\_2,3-reductase | BGC0000092 | Megalomicin\_biosynthetic\_gene\_cluster | 31 | 49.2877492877 | 78 | 1.9e-14 |
| AFP87517.1 | oxidoreductase | BGC0001159 | Marinopyrrole\_biosynthetic\_gene\_cluster | 30 | 47.5783475783 | 61 | 1.9e-09 |
